# Application of black phosphorus nanodots to live cell imaging

**DOI:** 10.1186/s40824-018-0142-x

**Published:** 2018-10-04

**Authors:** Yong Cheol Shin, Su-Jin Song, Yu Bin Lee, Moon Sung Kang, Hyun Uk Lee, Jin-Woo Oh, Dong-Wook Han

**Affiliations:** 1 Research Center for Energy Convergence Technology, Pusan National University, Busan, 46241 Republic of Korea; 2 Department of Cogno-Mechatronics Engineering, College of Nanoscience & Nanotechnology, Pusan National University, Busan, 46241 Republic of Korea; 3 Advanced Nano-Surface Research Group, Korea Basic Science Institute (KBSI), Daejeon, 34133 Republic of Korea; 4 Department of Nanofusion Technology, College of Nanoscience & Nanotechnology, Pusan National University, Busan, 46241 Republic of Korea

**Keywords:** Black phosphorus, Cell imaging, C2C12 myoblast, Fluorescence probe, Biomedical imaging

## Abstract

**Background:**

Black phosphorus (BP) has emerged as a novel class of nanomaterials owing to its unique optical and electronic properties. BP, a two-dimensional (2D) nanomaterial, is a structure where phosphorenes are stacked together in layers by van der Waals interactions. However, although BP nanodots have many advantages, their biosafety and biological effect have not yet been elucidated as compared to the other nanomaterials. Therefore, it is particularly important to assess the cytotoxicity of BP nanodots for exploring their potentials as novel biomaterials.

**Methods:**

BP nanodots were prepared by exfoliation with a modified ultrasonication-assisted solution method. The physicochemical properties of BP nanodots were characterized by transmission electron microscopy, dynamic light scattering, Raman spectroscopy, and X-ray diffractometry. In addition, the cytotoxicity of BP nanodots against C2C12 myoblasts was evaluated. Moreover, their cell imaging potential was investigated.

**Results:**

Herein, we concentrated on evaluating the cytotoxicity of BP nanodots and investigating their cell imaging potential. It was revealed that the BP nanodots were cytocompatible at a low concentration, although the cell viability was decreased with increasing BP nanodot concentration. Furthermore, our results demonstrated that the cells took up the BP nanodots, and the BP nanodots exhibited green fluorescence.

**Conclusions:**

In conclusion, our findings suggest that the BP nanodots have suitable biocompatibility, and are promising candidates as fluorescence probes for biomedical imaging applications.

## Background

Recently, various types of nanomaterials, such as carbon nanotube, graphene, quantum dot, and gold nanoparticle, have been proposed for a number of biomedical applications due to their distinct properties [1–5]. Among many nanomaterials, black phosphorus (BP) has emerged as a novel class of nanomaterials owing to its unique optical and electronic properties [6, 7]. BP can be readily exfoliated into nanodots because phosphorenes are stacked together in layers within bulk BP by weak van der Waals interactions [8–10]. The bandgap of exfoliated BP can be tailored according to the number of its layers (~ 0.3 eV for bulk BP and ~ 2 eV for single-layer BP) [7, 11, 12]. In addition, BP possesses superior electronic properties, particularly its high carrier mobility (in the order of 10^5^ cm^2^/V/s) and relatively high lattice thermal conductivity (12.1 W/mK) [13–16]. The uinique puckered structure of BP can also contribute the unusual mehcanical performance, such as a negative Poisson’s ratio, and unique optical properties, which show applied strain-dependent and anisotropic optical absorption spectrum [17–21]. Such unique optical and electronic properties of nano-sized BP, including an accurate optical-response property, anisotropic charge transport and semiconducting property with a layer-dependent bandgap, enable it to be employed for biomedical imaging applications [6, 10, 22]. In addition, the BP can be tailored by a variety of surface coating ligands, and it allows BP to be utilized as novel biomaterials. It has been revealed that the PEGylated BP nanosheets are internalized into cells by macropinocytosis or caveolae-dependent endocytosis pathways, and can be used as a 2D delivery platform for cancer theranostics [10]. In addition, PEGylated BP has been found to be photostable and suitable for photothermal therapy agent as well as photoacoustic imaging because it can convert near-infrared light to heat [19]. Additionally, there have been intensive efforts to employ BP in many widespread applications, such as lithium-ion rechargeable batteries, transistors, optoelectronic materials, and energy harvesting [22–25]. However, not much is known about the cytotoxicity and biological effects of BP as compared to the other nanomaterials, although BP nanodots have outstanding optical, electrical and mechanical properties. Therefore, prior to exploring their potential as novel nanomaterials, it is particularly important to assess the biosafety and biological effects of BP nanodots.

On the other hand, cell imaging is one of the highest priority for biomedical applications to fundamentally understand the biological basis of nanomaterials as well as to observe cellular behaviors. Hence, much research has been devoted to developing biofunctional imaging agents using various nanomaterials, such as quantum dots, gold nanoparticles and magnetic particles, that can be simultaneously used as diagnostic imaging and therapeutic agents [26–30]. However, the development of ideal nanomaterial-based imaging and therapeutic agents is still a challenge. In recent years, BP nanodots have attracted increasing attention in the field of biomedical imaging because of their unique optical and fluorescent characteristics [6, 19, 31, 32]. Meanwhile, the biological effects of BP nanodots, including cytotoxicity issues on mammalian cells, still remain to be addressed.

Herein, we focused on the cytotoxicity of BP nanodots against mammalian cells and further investigated their cell imaging potential. BP nanodots were prepared by exfoliation with a modified ultrasonication-assisted solution method [6]. The physicochemical properties of BP nanodots were characterized by transmission electron microscopy (TEM), dynamic light scattering, Raman spectroscopy, and X-ray diffraction (XRD). In addition, the cytotoxicity of BP nanodots against C2C12 skeletal myoblasts was determined by cell counting kit-8 (CCK-8) assay. Furthermore, the fluorescence analysis of C2C12 myoblasts treated with BP nanodots was conducted in order to explore their potential as biocompatible fluorescence probes for applications to cell and biomedical imaging.

## Methods

### Preparation of BP nanodots

BP nanodots were obtained by exfoliation with a modified ultrasonication-assisted solution method according to the procedure previously described [6, 33]. Briefly, BP (0.4 g, 12.8 mmol) was dispersed in deionized water by ultrasound sonication for 30 min to form several-layered BP nanodots. The 10 mL supernatant of BP suspension was transferred in fresh deionized water, and ultrasound sonicated for 10 min. These steps were repeated three times, and finally, BP nanodots were obtained.

### Characterizations of BP nanodots

The morphology of the BP nanodots was examined by TEM (H-7600, Hitachi, Tokyo, Japan) at an accelerating voltage of 80 kV. The Raman spectrum of BP nanodots was obtained by a Raman spectrometer (Micro Raman PL Mapping System, Dongwoo Optron Co., Kwangju, Korea) with excitation at 514.5 nm using an Ar-ion laser with a radiant power of 5 mW. The average hydrodynamic size and zeta potential of BP nanodots were analyzed using a Zetasizer (Malvern Instruments, Nano ZS, Worcestershire, UK). The XRD pattern of BP nanodots was obtained using the X-ray diffractometer (Empyrean series 2, PANalytical, Almelo, Netherlands) with Cu-Kα radiation (λ = 0.154 nm) at 40 kV and 30 mA. The measurements were conducted in the 2θ range of 10–80° with a scan rate of 2°/min at room temperature.

### Cytotoxicity evaluation of BP nanodots in C2C12 skeletal myoblasts

The C2C12 mouse skeletal myoblasts were purchased from the American Type Culture Collection (Rockville, MD, USA) and routinely maintained in Dulbecco’s modified Eagle’s Medium (DMEM, Welgene, Daegu, Korea) supplemented with 10% fetal bovine serum (Welgene) and a 1% antibiotic-antimycotic solution (containing 10,000 units penicillin, 25 μg amphotericin B and 10 mg streptomycin per mL, Sigma-Aldrich Co., Saint Louis, MO, USA) at 37 °C in a humidified atmosphere containing 5% CO_2_.

To assess the cytotoxicity of BP nanodots in the C2C12 skeletal myoblasts, a CCK-8 assay (Dojindo, Kumamoto, Japan) was conducted according to the manufacturer’s instructions. The number of viable cells was found to be directly proportional to the metabolic reaction products obtained in the CCK-8 assay [34, 35]. Briefly, the C2C12 myoblasts were seeded at a density of 5 × 10^4^ cells/mL on 24-well plates and incubated for 24 h. After then, BP nanodots were added with increasing concentrations (0 to 250 μg/mL) to the culture media, and the cells were further cultured for 24 and 48 h. Subsequently, the cells were incubated with a CCK-8 solution for an additional 2 h at 37 °C in the dark. The absorbance values were measured at 450 nm using a SpectraMax® 340 plate reader (Molecular Devices, Sunnyvale, CA, USA). The relative cell viability was determined as the percentage of the absorbance value in the cells to the absorbance value of a control group.

### Fluorescence imaging of C2C12 skeletal myoblasts

For fluorescence imaging of C2C12 skeletal myoblasts treated with BP nanodots, the cells were seeded at a density of 5 × 10^4^ cells/mL on 24-well plates and incubated for 24 h. The culture media were then replaced with fresh media containing BP nanodots at a predetermined concentration (0.5 μg/mL). To observe the internalization and fluorescence of BP nanodots in C2C12 skeletal myoblasts, the cells were incubated for 10 min, 30 min, 1 h, 2 h, 6 h, and 24 h. Subsequently, the cells treated with BP nanodots were thoroughly washed with Dulbecco’s phosphate-buffered saline (SigmaeAldrich Co.) and fixed with 3.7% formaldehyde solution (Sigma-Aldrich Co.) for 10 min. After fixation, the nucleus was stained using propidium iodide (PI, 1 μM, Sigma-Aldrich Co.) solution for 15 min, and the cells were imaged with an inverted fluorescence microscope (IX81, Olympus Optical Co., Osaka, Japan).

### Statistical analysis

All variables were tested in three independent cultures for each experiment, which was repeated twice (*n* = 6). The quantitative data are expressed as the mean ± standard deviation (SD). The data were tested for the homogeneity of the variances using the test of Levene, prior to statistical analysis. Statistical comparisons were carried out using a one-way analysis of variance (ANOVA; SAS Institute Inc., Cary, NC, USA), followed by a Bonferroni test for multiple comparisons. A value of *p* < 0.05 was considered statistically significant.

## Results and discussion

### Characterizations of BP nanodots

**Fig. 1:**
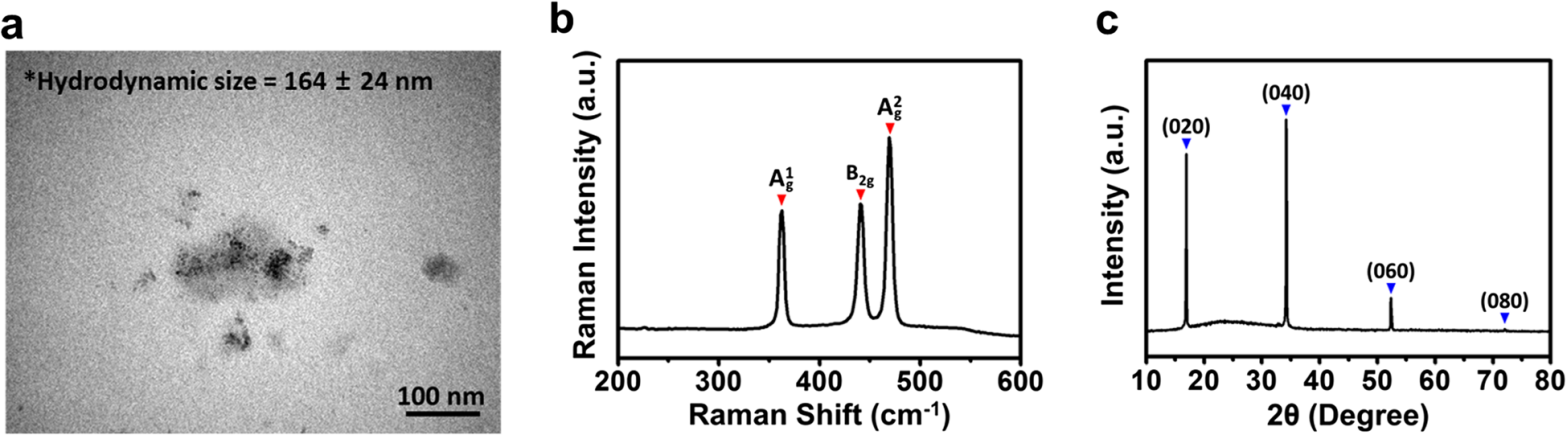
Characterizations of BP nanodots. **a** TEM image, **b** Raman spectrum, **c** XRD pattern of BP nanodots

The morphology of BP nanodots was observed by TEM and presented in Fig. 1a. TEM image showed the uniform spherical morphology of BP nanodots with the diameter of about several nanometers. In addition, it was shown that fine BP nanodots were clustered together into random agglomerates varying from a few nanodots to a few dozen nanodots because the freshly exfoliated BP nanodots were suspended in aqueous solution. Considering the cell culture condition, although TEM is a highly accurate technique for the characterization of nano-sized particles, their hydrodynamic size and surface charge should be considered because they would be suspended in culture media [36]. The average hydrodynamic size and zeta potential of BP nanodots were determined by dynamic light scattering and found to be 164 ± 24 nm and − 12.48 ± 0.66 mV, respectively. Therefore, it was indicated that the BP nanodots are successfully prepared from bulk BP and stably dispersed in aqueous solution, even though the hydrodynamic size of BP nanodots was slightly bigger than their individual size observed from TEM image.

To further confirm the successful preparation of BP nanodots, Raman spectroscopy was conducted because the BP typically exhibits specific bands, such as ${\mathrm{A}}_{\mathrm{g}}^1$, ${\mathrm{A}}_{\mathrm{g}}^2$ and B_2g_ modes of phosphorene [24, 37, 38]. Figure 1b shows the Raman spectrum of BP nanodots. The noticeable bands were observed at 362, 440 and 469 cm^− 1^, which were attributed to the ${\mathrm{A}}_{\mathrm{g}}^1$, ${\mathrm{A}}_{\mathrm{g}}^2$ and B_2g_ modes of phosphorene as reported in several previous studies [24, 37, 38]. In addition, the sharp Raman features in the spectrum implied that the BP nanodots were unique orthorhombic crystalline phosphorus structure. The orthorhombic crystalline structure of BP nanodots was also confirmed by XRD pattern (Fig. 1c). The XRD pattern of BP nanodots was found to be consistent with that of standard pattern of orthorhombic BP (JCPDS No. 76–1957). The characteristic diffraction peaks were observed at 16.9°, 34.2°, 52.3°, and 72.0°, corresponding to the d_020_ = 5.24 Å, d_040_ = 2.62 Å, d_060_ = 1.75 Å, and d_080_ = 1.31 Å, respectively. These results demonstrated that the BP nanodots employed in the present study were successfully prepared [24]. On the other hand, the nanometer-scale diameter of BP nanodots can greatly facilitate the interactions between BP nanodots and cells. However, the BP nanodots having nanometer-scale diameter might exhibit undesirable toxic effects on cells and tissues that have not been found in bulk ones. Therefore, we evaluated the cytotoxic effects on C2C12 murine skeletal myoblasts, prior to exploring their cell imaging potentials.

### Cytotoxicity of BP nanodots against C2C12 skeletal myoblasts

**Fig. 2:**
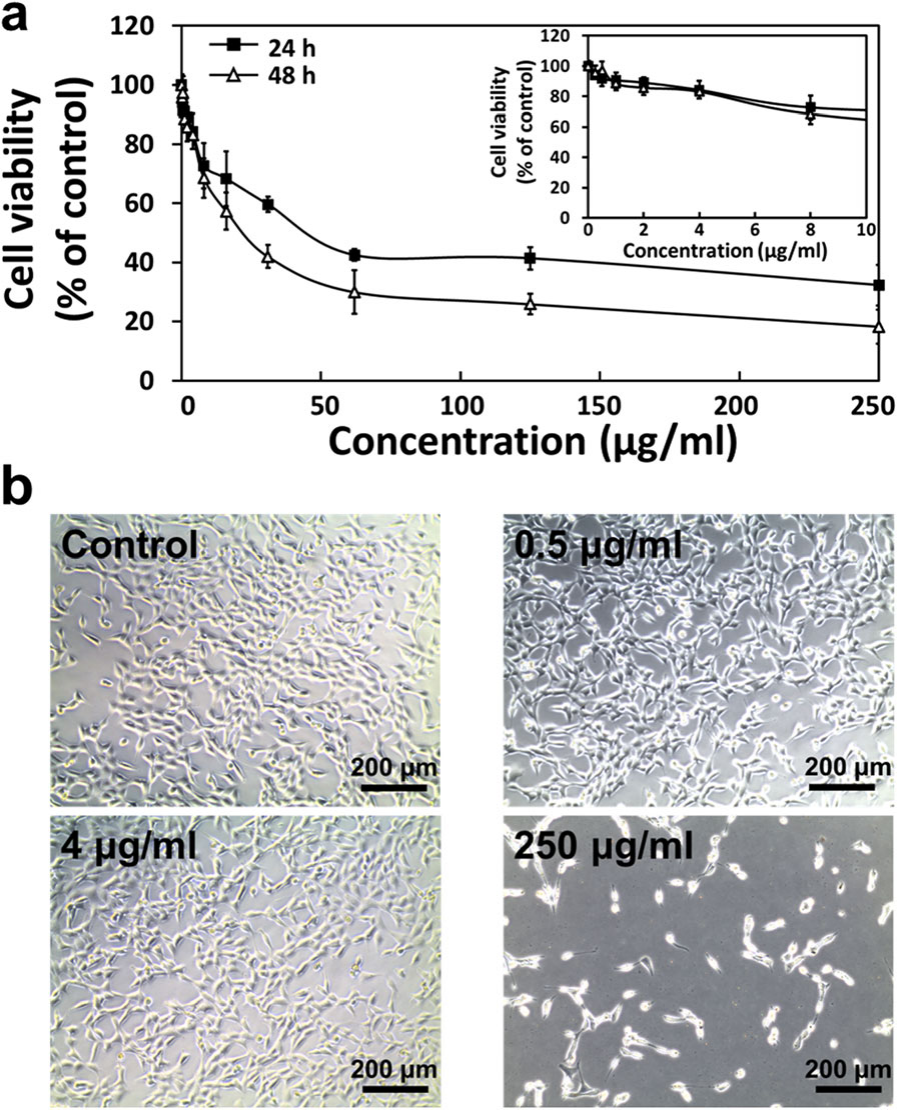
Cytotoxicity of BP nanodots against C2C12 skeletal myoblasts. **a** C2C12 skeletal myoblast viability after the 24 (■) and 48 h (△) of incubation with various concentrations of BP nanodots (0 to 250 μg/mL). **b** Representative optical microscopic images of C2C12 skeletal myoblasts cultured with BP nanodots (0, 0.5, 4, and 250 μg/mL) for 24 h. The viability of C2C12 myoblasts was determined using a CCK-8 assay, and all photographs shown in this figure are representative of six independent experiments with similar results

The cell viability of C2C12 skeletal myoblasts was determined according to the concentrations of BP nanodots (0 to 250 μg/mL) by using CCK-8 assay, based on the metabolic activity of mitochondria, in order to examine their influence on mammalian cells. As presented in Fig. 2a, the myoblast viability was decreased with increasing BP nanodot concentrations. The cell viability was significantly (*p* < 0.05) decreased at concentrations higher than 10 μg/mL after 24 h of incubation with BP nanodots, and it was approximately 30% of the control at the highest concentration (250 μg/mL). Meanwhile, over 84% of C2C12 myoblasts were viable at a concentration of 4 μg/mL. The cytotoxicity of BP nanodots after 48 h also dose-dependently increased. In addition, after longer time periods of incubation with BP nanodots (48 h), the decrease in cell viability was more significant at concentrations higher than 10 μg/mL. However, the cell viability was ~ 83% at 4 μg/mL of BP nanodots, indicating that the BP nanodots showed little cytotoxicity at low concentrations (≤ 4 μg/mL). These results are in contradiction with several previous studies, which showed that there was no observable cytotoxicity [6, 39, 40]. In particular, BP nanodots were reported to show little cytotoxicity even at 1000 μg/mL to HeLa cell (human cervical carcinoma line), COS-7 cell (fibroblast-like monkey kidney cell line) and CHO-K1 cell (chinese hamster ovary cell) [6]. This can be attributed to the fact that nanomaterials and their derivatives exhibit cell type specific toxicity [41–44]. In the present study, BP nanodots showed significant cytotoxicity against C2C12 mouse skeletal myoblasts at high concentrations (< 10 μg/mL).

The morphological observations further support the cytotoxicity evaluation results. The morphologies of C2C12 skeletal myoblasts treated with each concentration of BP nanodots were observed by optical microscope, and the representative optical images (0, 0.5, 4, and 250 μg/mL of BP nanodots) were shown in Fig. 2b. The cells were well grown at concentrations lower than 4 μg/mL, and there was no morphological signs of cytotoxicity. However, at a concentration of 250 μg/mL BP nanodots, cells showed shrunken morphology, and the number of cells was significantly decreased. These results were in good accordance with the cytotoxicity profile (Fig. 2a). According to the previous literature, the cytotoxicity of nanomaterials, such as carbon nanotube, graphene and gold nanoparticles, is closely related to the intracellular reactive oxygen species (ROS) production and membrane disruption [34, 36, 45]. The cytotoxicity of BP nanodots can also have relevance to both intracellular ROS production and cell membrane disruption [36, 40]. Meanwhile, it has been widely documented that the effects of nanomaterials are highly dependent on their concentration, size, shape, and surface charge [34–36, 46–50]. Hence, it is particularly desirable to use nano-sized BP having proper size, shape and surface charge. A series of previous studies indicate that the negatively charged particles show lower cytotoxicity than positively charged particles [47–50], and the cellular uptake of rod-shaped particles is lower than their spherical counterparts [51–53]. In addition, recent studies show that the larger BP particles (~ 884 nm) have more cytotoxic effects on cells compared to the smaller ones (~ 209 nm) [6, 10, 40, 54]. However, there have been also many conflicting results in the literature that may be due to variations in many other factors, such as size, particle dimension and surface functional moiety, and the effects of those factors on the cytotoxicity of BP particles may be only valid when comparing several different types of BP. The BP nanodots, used in the present study, exhibited cytotoxicity against C2C12 myoblasts in a dose-dependent manner, but not at low concentrations (≤ 4 μg/mL), as consistent with previous studies that have documented that BP has good biocompatibility and can be applicable to biomedical applications, such as photoacoustic imaging and drug delivery system [6, 10, 19, 36, 54]. Therefore, it is suggested that BP nanodots at low concentrations have a suitable biocompatibility for their use in biomedical applications.

### In vitro fluorescence imaging of BP nanodots

**Fig. 3:**
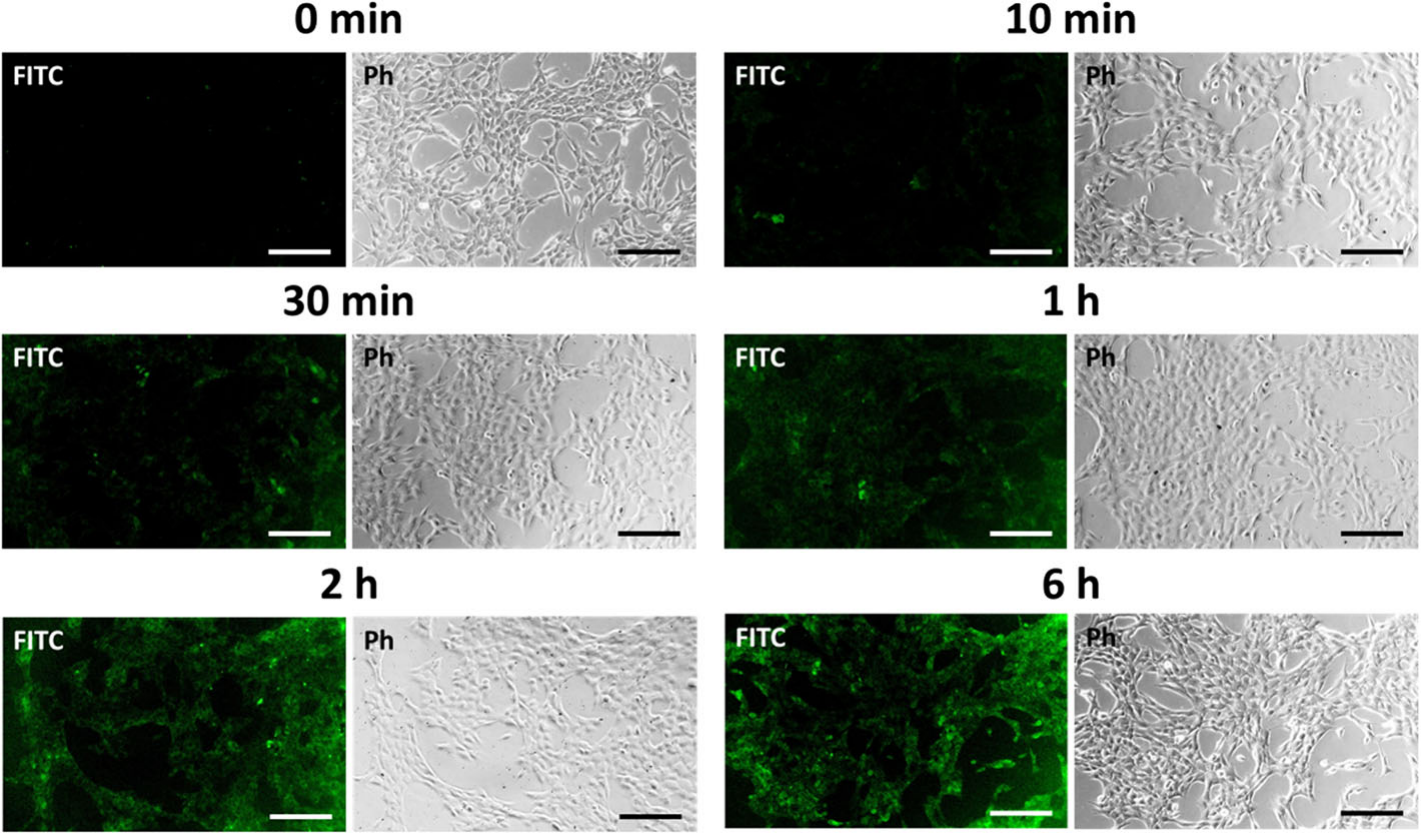
Time-lapse fluorescence imaging of BP nanodots. Time-lapse fluorescence images of C2C12 myoblasts cultured with 0.5 μg/mL of BP nanodots for 10 min, 30 min, 1 h, 2 h, and 6 h. The scale bars are 200 μm

To explore the potential of BP nanodots as a novel cell imaging agent, in vitro fluorescence imaging was carried out. Considering the cytotoxicity evaluation results (Fig. 2), we cultured C2C12 myoblasts with the 0.5 μg/mL of BP nanodots and analyzed by fluorescence microscopy. Figure 3 shows the fluorescence images of C2C12 myoblasts cultured with BP nanodots for 10 min, 30 min, 1 h, 2 h, and 6 h.

**Fig. 4:**
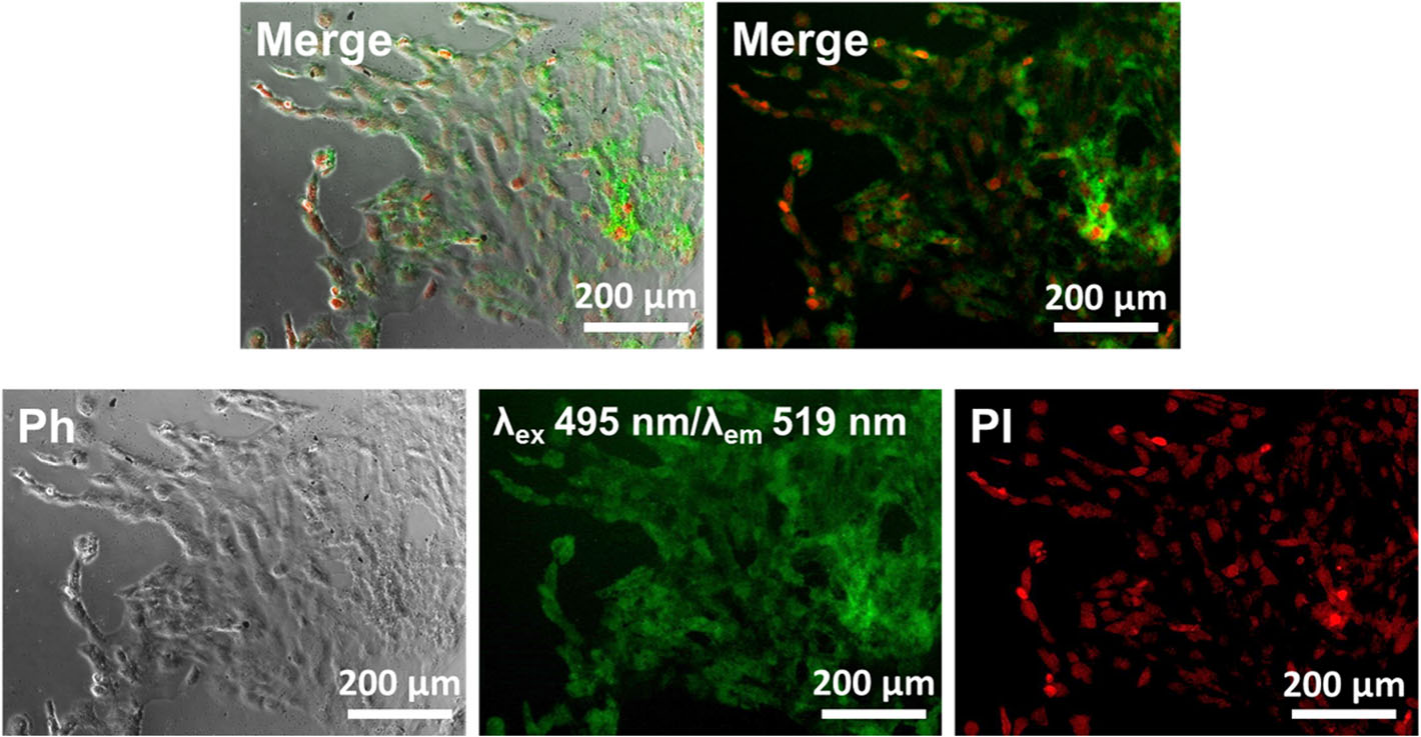
In vitro fluorescence imaging of BP nanodots. Fluorescence images of C2C12 skeletal myoblasts treated with 0.5 μg/mL of BP nanodots for 24 h. Cell nuclei were counterstained with PI (red). All photographs shown in this figure are representative of six independent experiments with similar results

The fluorescence images showed that the BP nanodots were internalized into cells as incubation time increased from 0 to 6 h. It has been reported that the nano-sized BP can be internalized into cells via either macropinocytosis or caveolae-dependent endocytosis, and then transported through endosome and lysosome, followed by lysosomal degradation [10]. Moreover, the negatively charged surface of BP nanodots (− 12.48 ± 0.66 mV) can also facilitate their internalization into cells via caveolae-dependent endocytosis [49, 50]. Therefore, BP nanodots were also able to enter cells via macropinocytosis or caveolae-dependent endocytosis pathways. After 30 min of incubation with BP nanodots, the BP nanodots were accumulated in the cytoplasm, and the green fluorescence of BP nanodots was detected. Then, after more time elapsed, more BP nanodots could be internalized into cells, and accumulated in the cytoplasm, resulting in a clear green fluorescence from C2C12 myoblasts. After 6 h of incubation, BP nanodots could be effectively internalized into C2C12 myoblasts, and a strong green fluorescence was exhibited from BP nanodots concentrated in myoblasts. To clearly verify the fluorescence imaging potential of BP nanodots, the fluorescence images were taken after allowing sufficient time (24 h) for the BP nanodots to be internalized into cells (Fig. 4).

As shown in Fig. 4, the cells were favorably grown with 0.5 μg/mL BP nanodots showing typical spindle-like morphology. Notably, during the culture period, no observable cytotoxicity was detected. Moreover, the strong green fluorescence was exhibited from the BP nanodots in the cytoplasm of C2C12 myoblasts. It has been acknowledged that the graphene-based nanomaterials or transition-metal dichalcogenides, such as WS_2_ and MoS_2_, are able to serve as a platform for biomedical imaging [55–60]. However, such graphene- or transition-metal dichalcogenide-based nanomaterials are not biochemically degradable, which in turn, it has been largely restricted to their application for biomedical applications due to inadequate clearance from the organs and tissues [6, 61–64]. On the other hand, the BP nanodots can be biochemically degraded by the internalization into cells via macropinocytosis or caveolae-dependent endocytosis pathways, followed by lysosomal degradation, and favorably cleared from organs and tissues during blood circulation [6, 10, 65]. BP nanodots have been recently known to not only have unique optical and electronic properties but to also possess specific fluorescence properties.

The BP nanomaterials can exhibit blue- and green-emission (λ_em_ = 461 and 519 nm) under ultraviolet (UV) and visible light excitation (λ_ex_ = 358 and 495 nm), respectively [6]. However, the UV light has been extensively recognized to induce significantly optical damages by activating apoptosis signaling pathway as well as the production of ROS [66–69]. Hence, we observed myoblasts treated with BP nanodots under visible excitation (λ_ex_ = 495 nm and λ_em_ = 519 nm). As revealed in Fig. 4, the BP nanodots were successfully internalized into C2C12 myoblasts, concentrated in their cytoplasm, and exhibited a specific green fluorescence without undesirable cytotoxic effects. These results implied that the BP nanodots can be used as novel intrinsic fluorescence probes for biomedical applications. In addition, recent studies have revealed that the nano-sized BP have promising potential as drug delivery carrier, photothermal/photodynamic therapy agent and photodetector [6, 8, 10, 31, 54, 65]. Moreover, BP nanodots can be functionalized with various surface groups tailored for target applications, such as drug delivery, photothermal/photodynamic therapy and photodetection, thus allowing the BP nanodots to be a promising candidate as novel biofunctional materials for biomedical applications. However, the more detailed biological effects of BP nanodots are still largely unknown, and further comprehensive studies should be performed before their clinical applications. Nonetheless, our preliminary studies on the potential of BP nanodots suggest that the BP nanodots can be employed as promising fluorescence probes for applications to cell and biomedical imaging.

## Conclusions

This study was designed to evaluate the cytotoxicity of BP nanodots and to explore their potential for cell imaging. We successfully prepared BP nanodots by exfoliation using a modified ultrasonication-assisted solution method. The BP nanodots were found to have a nanoscale-size, indicating that the BP nanodots consisted of several layers of BP. In addition, from the cytotoxicity evaluation using C2C12 skeletal myoblasts, it was revealed that the BP nanodots showed a dose-dependent cytotoxicity, and were cytocompatible at low concentrations (≤ 4 μg/mL). Moreover, our results demonstrated that the BP nanodots could be easily internalized into C2C12 skeletal myoblasts, and exhibited green fluorescence under visible light excitation without undesirable cytotoxic effects. Our findings suggest that the BP nanodots have a suitable biocompatibility, and are promising candidates as fluorescence probes for biomedical imaging applications, although further comprehensive studies with BP nanodots are needed to employ the BP nanodots for in vivo and clinical applications.

## Abbreviations

ANOVA: Analysis of variance
BP: Black phosphorus
CCK-8: Cell counting kit-8
DMEM: Dulbecco’s modified Eagle’s Medium
PI: Propidium iodide
ROS: Reactive oxygen species
SD: Standard deviation
TEM: Transmission electron microscopy
UV: Under ultraviolet
XRD: X-ray diffraction
